# *cytomapper*: an R/Bioconductor package for visualization of highly multiplexed imaging data

**DOI:** 10.1093/bioinformatics/btaa1061

**Published:** 2020-12-26

**Authors:** Nils Eling, Nicolas Damond, Tobias Hoch, Bernd Bodenmiller

**Affiliations:** Department of Quantitative Biomedicine, University of Zurich, 8057 Zurich, Switzerland; Institute for Molecular Health Sciences, ETH Zurich, 8093 Zurich, Switzerland; Department of Quantitative Biomedicine, University of Zurich, 8057 Zurich, Switzerland; Institute for Molecular Health Sciences, ETH Zurich, 8093 Zurich, Switzerland; Department of Quantitative Biomedicine, University of Zurich, 8057 Zurich, Switzerland; Institute for Molecular Health Sciences, ETH Zurich, 8093 Zurich, Switzerland; Department of Quantitative Biomedicine, University of Zurich, 8057 Zurich, Switzerland; Institute for Molecular Health Sciences, ETH Zurich, 8093 Zurich, Switzerland

## Abstract

**Summary:**

Highly multiplexed imaging technologies enable spatial profiling of dozens of biomarkers *in situ*. Here, we describe *cytomapper*, a computational tool written in R, that enables visualization of pixel- and cell-level information obtained by multiplexed imaging. To illustrate its utility, we analysed 100 images obtained by imaging mass cytometry from a cohort of type 1 diabetes patients. In addition, *cytomapper* includes a Shiny application that allows hierarchical gating of cells based on marker expression and visualization of selected cells in corresponding images.

**Availability and implementation:**

The *cytomapper* package can be installed via https://www.bioconductor.org/packages/release/bioc/html/cytomapper.html. Code for analysis and further instructions can be found at https://github.com/BodenmillerGroup/cytomapper_publication.

**Supplementary information:**

Supplementary data are available at *Bioinformatics* online.

## 1 Introduction

Immunohistochemistry (IHC) and immunofluorescence (IF) are common approaches for visualization of proteins in tissues. Highly multiplexed IHC and IF methods have recently been developed to increase the number of proteins being measured in parallel (Gerdes *et al.*, 2013; Huang et al., 2013). Multiplexing using antibodies labelled with fluorescent dyes, oligonucleotides or metal tags allows high-resolution imaging of tens of proteins simultaneously (Angelo *et al.*, 2014; Giesen *et al.*, 2014; Goltsev *et al.*, 2018; Lin et al., 2018; Saka et al., 2019).

One of the latter approaches is imaging mass cytometry (IMC), during which tissues are stained using metal-conjugated antibodies (Giesen *et al.*, 2014). After data acquisition, raw output files are processed to create multi-channel images and segmentation masks. This enables the extraction of cell-specific measurements, such as mean ion counts per marker and morphological features (Damond *et al.*, 2019). Custom scripts (Jackson et al., 2020; Keren et al., 2018), image analysis software such as *CellProfiler* (Carpenter *et al.*, 2006), and specialized tools based on graphical user interfaces (GUIs) (Schapiro et al., 2017; Somarakis et al., 2019; Stoltzfus et al., 2020) are used to process and analyse high-dimensional spatial expression data.

Here, we combine the image and single-cell data analysis capabilities of Bioconductor (Gentleman *et al.*, 2004) to allow visualization of pixel- and cell-level information obtained by highly multiplexed imaging technologies such as IMC. The R/Bioconductor package *cytomapper* allows high flexibility in terms of image manipulation (e.g. transformations), integrates with common single-cell data analysis strategies (e.g. cell phenotyping), and includes a Shiny application to enable hierarchical gating and visualization of selected cells. We demonstrate the utility of *cytomapper* by using it for biological exploration of type 1 diabetes progression and quality control of segmentation results.

## 2 Results

**Fig. 1.**
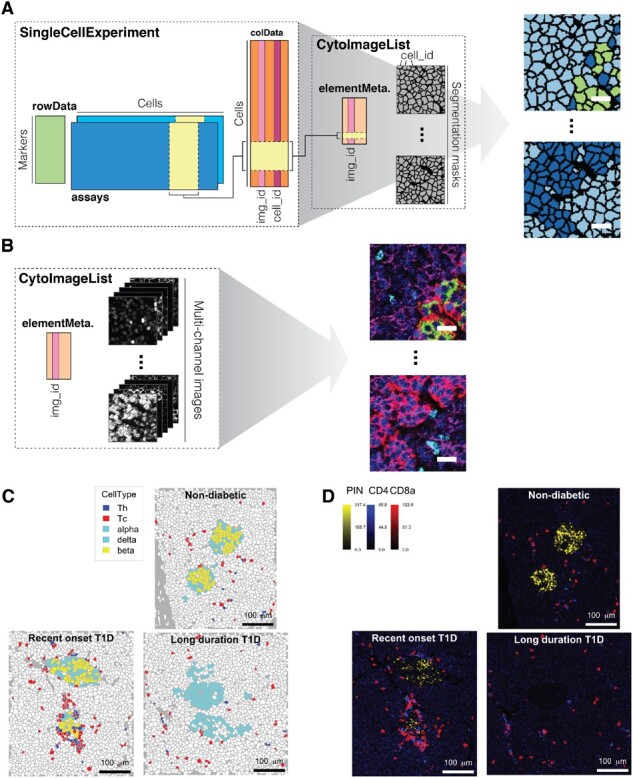
*cytomapper* functionality. (**A**) The *plotCells* function combines a *SingleCellExperiment* and *CytoImageList* object to visualize marker expression or cell-specific metadata on segmentation masks (Supplementary Notes S1.4 and S1.5). (**B**) The *plotPixels* function requires a *CytoImageList* object to visualize the combined expression of up to six markers as composite images (Supplementary Note S1.6). Scale bars: 20 µm. (**C, D**) For each condition (healthy, recent onset and long-duration T1D), images with the highest density of cytotoxic and helper T cells were selected. (C) The *plotCells* function colours selected cells (islet cells, cytotoxic and helper T cells) by their cell type and leaves all other cells white. (D) Proinsulin (PIN) in yellow marking β cells, CD4 in blue marking helper T cells and CD8a in red marking cytotoxic T cells are visualized as composite images by merging pixel-level information. Raw pixel-intensities were multiplied by 10, 8 and 10 for PIN, CD4 and CD8a, respectively to increase the contrast of the images. Scale bars: 100 µm

Single-cell expression values and cell-specific metadata such as cell type information are stored in a *SingleCellExperiment* class object (Amezquita *et al.*, 2020) (Fig. 1A). The *cytomapper* package provides the *CytoImageList* container that stores single- or multi-channel images (Supplementary Note S1.2 and Fig. 1A and B). These objects contain segmentation masks represented as single-channel images; or multi-channel images where each channel contains pixel intensities of an individual marker. By providing information regarding a cell’s object identifier and a unique image name, the *plotCells* function colours segmentation masks by marker expression or cell-specific metadata (Fig. 1A). Multi-channel images are visualized as composites of up to six channels using the *plotPixels* function (Fig. 1B).

To demonstrate the functionality of the *cytomapper* package we used it to visualize type 1 diabetes (T1D) samples acquired by IMC (Supplementary Note S1.1). T1D is characterized by β cell loss caused by autoreactive immune cell infiltration (Atkinson *et al.*, 2014) and we previously imaged pancreatic samples from patients with recent-onset and long-duration, as well as healthy controls. We ranked images based on the density of cytotoxic and helper T cells and selected the image with highest density per condition. Using the *cytomapper* package, we visualized all islet cell types, and cytotoxic and helper T cells in selected images (Fig. 1C). To visually confirm cell phenotypes, we further displayed cell type specific markers [proinsulin (PIN): β cells; CD4: helper T cells; CD8a: cytotoxic T cells] as composite images (Fig. 1D). By visualizing selected images, we observe, as expected, that (i) β cells and proinsulin expression are lost during T1D progression and (ii) T cells invade the microenvironment during early onset of T1D (Damond *et al.*, 2019). The *cytomapper* package also allows the visualization of tens to hundreds of images in parallel. As described in Supplementary Note S2.1 and Supplementary Figures S1 and S2, the loss of β cells and reduction of PIN expression was observed across 100 selected images from the full set of 845 images acquired (Damond *et al.*, 2019).

Segmentation and labelling of cell phenotypes are essential steps of most multiplexed imaging pipelines. The *cytomapper* package provides function settings to outline cells on composite images based on their segmentation results. Furthermore, outlines can be coloured based on cell-specific metadata, such as cell type information (Supplementary Fig. S3). This visual quality control step is recommended prior to downstream analyses such as clustering or the testing of associations with clinical data.

Cell phenotyping is commonly performed by clustering and cluster annotation. However, a number of classification strategies have recently been developed to label cells based on a given reference (Abdelaal *et al.*, 2019). To facilitate cell labelling, we developed the *cytomapperShiny* function, which opens a Shiny GUI that allows hierarchical gating on the expression levels of up to 24 markers. Selected cells are either visualized as coloured objects on segmentation masks or as outlines on composite images (Supplementary Fig. S4). Furthermore, selected cells can be downloaded in form of a *SingleCellExperiment* object for use in downstream processes such as training and cell type classification. The ease of generation and improved quality of training data enabled by this function will meet the growing demand for supervised classification methods (Abdelaal *et al.*, 2019).

## 3 Conclusion

The *cytomapper* package offers a set of functions to visualize cell- and pixel-level information obtained using highly multiplexed imaging technologies across tens to hundreds of images. We demonstrated the use of *cytomapper* with IMC data. However, data obtained using other multiplexed imaging technologies such as MIBI (Angelo *et al.*, 2014), 4i (Gut *et al.*, 2018), t-CyCIF (Lin *et al.*, 2018) and CODEX (Goltsev *et al.*, 2018) could be visualized using the *cytomapper* package. The only requirements are single-cell read-outs, multi-channel tiff stacks and/or segmentation masks. By using the *SingleCellExperiment* object as data container, *cytomapper* integrates with an extensive set of single-cell data analysis tools as well as other R packages designed for spatial data analysis (Dries *et al.*, 2019; Yang et al., 2020). Finally, we provide the *SingleCellExperiment* and *CytoImageList* objects containing the presented data in form of the newly developed *imcdatasets* package on Bioconductor.

## Funding

This work was supported by the European Molecular Biology Organization [ALTF 1194-2019]; the JDRF [3-PDF-2020-937-A-N]; and a National Institute of Health grant [DK108132].

*Conflict of Interest*: none declared.

## Supplementary Material

btaa1061_Supplementary_Data
