# Supplementary material for: cytomapper: an R/Bioconductor package for visualization of highly multiplexed imaging data
Source: Bioinformatics. 2020 Dec 26;36(24):5706–8. doi: 10.1093/bioinformatics/btaa1061 (PMC8023672; doi:10.1093/bioinformatics/btaa1061)
Supplement: btaa1061_Supplementary_Data [file btaa1061_supplementary_data.pdf]

Supplementary Material for *cytomapper:* an R/Bioconductor package for visualisation of highly multiplexed imaging data

Nils Eling, Nicolas Damond, Tobias Hoch and Bernd Bodenmiller

S1 SUPPLEMENTARY NOTES AND METHODS 2
S1.1 Type 1 Diabetes dataset 2
S1.2 The CytoImageList container 3
S1.3 The SingleCellExperiment container 4
S1.4 Linking images and cell-specific data 5
S1.5 The plotCells function 5
S1.6 The plotPixels function 6
S1.7 Image normalisation 6
S1.8 Additional plotting parameters 7
S1.9 The Shiny application 8
S2 CASE STUDY 9
S2.1 β cell and proinsulin loss during T1D progression 9
S3 CODE AND DATA AVAILABILITY 10
S4 SUPPLEMENTARY FIGURES 11
S5 REFERENCES 15

S1 SUPPLEMENTARY NOTES AND METHODS

S1.1 Type 1 Diabetes dataset

We highlight the functionality of *cytomapper* by re-analysing a dataset acquired by imaging mass cytometry (IMC). We previously profiled 1,581 pancreatic islets from 12 donors, 4 of which were non-diabetic, 4 had recently been diagnosed with Type 1 Diabetes (T1D) (< 0.5 years) and 4 had long-standing T1D (> 8 years) (Damond *et al.*, 2019). For this, we developed an antibody panel consisting of 35 markers, 2 nuclear stains and PD-1, which was excluded from analysis in the original paper, to profile the interactions between immune and pancreatic islet cells during disease progression. A total of 845 images were acquired where each image captures single or few pancreatic islets.

Cells and pancreatic islets were segmented using *CellProfiler* (Kamentsky *et al.*, 2011) and *ilastik* (Sommer *et al.*, 2011) by selecting informative channels (https://github.com/BodenmillerGroup/ImcSegmentationPipeline). Multi-channel tiff files were directly created using the *imctools* python package (https://github.com/BodenmillerGroup/imctools) (Damond *et al.*, 2019). *Ilastik* was further used to classify cells into four categories: islet, immune, exocrine and "other". Islet cells were further classified into α, β, δ and γ cells; immune cells were classified into B cells, cytotoxic and helper T cells, monocytes/macrophages, and neutrophils; exocrine cells were sub-divided into acinar and ductal cells and "other" cells were classified into endothelial, stroma and unknown (Damond *et al.*, 2019).

As an example dataset, we selected one representative donor per disease stage (non-diabetic, recent onset, long-duration) and randomly selected 100 images (33 non-diabetic, 33 recent-onset, 34 long-duration) from these three donors, containing a total of 252,059 cells.

A *SingleCellExperiment* object was created to store cell-specific expression values and metadata. Mean pixel intensities per cell and marker were loaded into the *counts assay* slot and arsinh-transformed counts were stored in the *exprs assay* slot. Cell-specific (e.g. cell identifier, cell type information, location, area, islet- and neighbour relationships), patient-specific (e.g. case identifier, disease stage, age,

gender) and image-specific (image identifier, slide identifier, dimensions) metadata were stored in the *colData* entry of the *SingleCellExperiment* object. Marker-specific metadata such as metal isotope, marker name, stock concentration and channel identifier were loaded into the *rowData* slot. Attention needs to be paid to the correct ordering of the channels and cells in the *SingleCellExperiment* object. For more information see the section "S1.3 The SingleCellExperiment container".

We used the *loadImages* function provided by *cytomapper* to read in the multi-channel tiff stacks and segmentation masks into *CytoImageList* objects. The correct image identifier was added to the metadata entry of both *CytoImageList* objects to link them to information stored in the *SingleCellExperiment* object. Furthermore, the *CytoImageList* object storing segmentation masks was scaled by a factor of 65535 to account for 16-bit scaling. In memory and on disk (as .rds file) storage specifications for the generated *SingleCellExperiment* and *CytoImageList* objects can be seen in Supplementary Table 1.

Supplementary Table 1: In memory and on disk storage specifications for *SingleCellExperiment* and *CytoImageList* objects

Object type	Size in memory	Size on disk (.rds file)
Multi-channel CytoImageList	7400 Mb	1780 Mb
Masks CytoImageList	200 Mb	8.6 Mb
SingleCellExperiment	248.6 Mb	145 Mb

S1.2 The CytoImageList container

The *CytoImageList* container provides an S4 class object to store multiple single- or multi-channel images. We created helper functions to get and set the channel names for multi-channel images and provide a number of functions for consistent subsetting. In places where segmentation masks are needed for visualisation, *cytomapper* will test if the supplied *CytoImageList* object only stores single-channel images that contain integer values or 0. The integer values indicate the numeric object identifier of each cell while 0 labels the background of the image. Furthermore, the *CytoImageList* container stores metadata for each image (e.g.

disease type) and supports easy subsetting. When using the *cytomapper* functionality, unique image identifiers must be provided in the *CytoImageList* metadata (see below).

The *CytoImageList* class supports storing images with different x- and y-dimensions in individual slots. However, each image needs to have the same z-dimension (same number of channels).

For convenience, we have created the *loadImages* function that reads a single image or multiple images (in .tiff, .png or .jpeg format) into R while creating a *CytoImageList* object. The *loadImages* function accepts a *pattern* argument to specifically read in a selection of images (matched by regular expressions). This approach facilitates the fast and easy visualisation of a subset of images without the need to read in all images into memory.

Users can apply all existing image analysis functions exported by the *EBImage* Bioconductor package directly to a *CytoImageList* object by using the *endoapply* function exported by the *S4Vectors* package.

S1.3 The SingleCellExperiment container

The *SingleCellExperiment* container is a popular S4 class used in Bioconductor workflows and tools to store information of individual cells (Amezquita *et al.*, 2020). The main slots of the object store raw and transformed expression data, cell-specific metadata, gene/marker-specific metadata and low-dimensional embeddings. Here, we store the mean pixel intensities per cell in the *counts* assay slot and the arsinh-transformed (using a co-factor of 1) mean pixel intensities in the *exprs* assay slot. The *counts* assay slot was chosen to reuse the most appropriate *assay* getter function exported by the *SingleCellExperiment* package. In this manuscript, the term “counts” refers to the assay slot storing mean ion counts per cell, which corresponds to mean pixel intensities per cell in the case of imaging mass cytometry data. However, any named assay slot can be visualised using the *cytomapper* package. The *cytomapper* package combines *CytoImageList* and *SingleCellExperiment* objects to visualise information contained in the *SingleCellExperiment* object on images contained in the *CytoImageList* object.

By storing cell-specific data in a *SingleCellExperiment* the user can apply the vast number of functions provided by Bioconductor to perform data analyses (e.g. dimensionality reduction, clustering, data visualisation, differential analysis).

S1.4 Linking images and cell-specific data

As explained above, the *CytoImageList* object contains multiple single- or multi-channel images. The unique image IDs need to be stored within the metadata of the object. In the case of segmentation masks, integer pixel values represent the cells' object identifiers. This information is used to link cells and images to data stored in the *SingleCellExperiment* object. Both, the unique image ID and cell ID, are stored in the *colData* slot of the *SingleCellExperiment* object and matching to the images stored in the *CytoImageList* object is done internally. In that way, the *SingleCellExperiment* object can be subsetted to only contain a specific selection of cells (**Fig. 1C, D**).

S1.5 The plotCells function

The *plotCells* function visualises cell-specific marker expression or metadata on segmentation masks. The main input to the *plotCells* function is a *CytoImageList* object containing the segmentation masks and a *SingleCellExperiment* object containing the cell-specific information, the image ID and the cell ID slot entries. Furthermore, the user can specify which markers or metadata to visualise, chose the colour scale per marker or metadata entry and which expression data slot to use.

The combined expression of up to six markers can be visualised on the segmentation masks using additive colour mixing. However, we do not recommend displaying multiple markers with overlapping expression patterns on the segmentation masks. The user can specify two or more colours for each marker that are interpolated to generate a marker-specific colour scale. When displaying marker expression on segmentation masks, colours are scaled between the minimum and maximum expression count across all cells contained in the *SingleCellExperiment* object. This is also true when subsetting images prior to plotting.

Furthermore, individual metadata entries such as cell phenotype can be visualised on the segmentation mask using automatic or manual colour selection. Cells can either be filled or outlined by metadata entries.

S1.6 The plotPixels function

The *plotPixels* function visualises marker expression by displaying a pseudo-colour representation of pixel-intensities. When visualising single channels, the *viridis* colour scale is used to display low intensities as blue and high intensities as yellow. Between two and six channels can be additively merged to display a pseudo-colour composite image. The default colours for displaying high intensities are red, green, blue, cyan, magenta and yellow. However, colours can be changed by providing at least two colours per channel (minimum and maximum) to generate a continuous colour scale. Colours are scaled between the minimum and maximum pixel-intensity across all displayed images. Therefore, when subsetting images before plotting, the range of pixel-intensities can change.

The user can control the brightness (b), contrast (c) and gamma value (g) of the displayed image by setting the *bcg* parameter. The *bcg* parameters used for the current figures are listed in the figure legends. Only the contrast parameter was changed in the presented analyses.

Furthermore, the user can provide an additional *SingleCellExperiment* and *CytoImageList* object containing segmentation masks (see above). By doing so, cells can be outlined based on metadata features stored in the *SingleCellExperiment* object (**Supplementary Fig. 3**).

S1.7 Image normalisation

The *EBImage* R/Bioconductor package (Pau *et al.*, 2010) provides a *normalize* function that scales pixel-intensities between 0 and 1 either channel-wise or across all channels. The user can further provide a clipping range to set pixel-intensities to either 0 or 1 if outside of the range. We have adapted this scaling normalization to multiple multi-channel images. By default, the *cytomapper normalize* function scales pixel

intensities channel-wise across all images contained in the *CytoImageList* object. This default setting is chosen to display staining differences between images (Somarakis *et al.*, 2019). The user can also choose to perform the scaling normalisation per image by setting *separateImages = TRUE*. We further provide a *scaleImages* function that multiplies the pixel intensities per image with a constant value. This is useful when read-in pixel intensities are not correctly scaled.

S1.8 Additional plotting parameters

The user can modify different features of the displayed images, save the images or get them returned in R for further analysis. These options are shared between the *plotPixels* and *plotCells* function and are documented under the key *plotting-param* in R. The colour of cells on the segmentation masks that are not contained in the *SingleCellExperiment* object can be set using the *missing_colour* parameter. The background colour can be changed by setting *background_colour*. The length, label, size, colour and position of the scale bar can be changed using the *scale_bar* parameter. Image titles can be controlled by setting the *image_title* parameter. All features of the colour legend can be controlled by setting the *legend* parameter. To save the displayed images, the user can specify the *save_plot* parameter. This takes a list containing the *filename* and a *scale* scalar x. The later scales the resolution of the image x fold. By setting *return_plot = TRUE* the displayed images including image titles and scale bars are returned as a single plot or list of plots. When setting *return_images = TRUE* a list of individual images is returned. However, scale bars and image titles are lost when returning composite images. By default, multiple images are plotted on a grid with varying margins between individual images depending on the maximum image width and height. To further increase the margin between individual images, the *margin* parameter can be set. The user can further set *display = “single”* to plot individual images in their own graphics device instead of on a grid. By default, each channel is scaled between its minimum and maximum before creating the composite image. This behaviour can be supressed and relative differences between channels can be observed by setting *scale = FALSE*. By default, pseudo-colours are interpolated between neighbouring pixels to smooth the image. Interpolation is supressed by setting *interpolate = FALSE*.

To suppress the display of the legend, image title and scale bar, their corresponding parameters can be set to *NULL.*

S1.9 The Shiny application

We developed an interactive application using the R packages *shiny* and *shinydashboard* to gate cells based on their expression values and to visualise selected cells on images. The *cytomapperShiny* function takes a *SingleCellExperiment* object storing cell-specific features and *CytoImageList* objects storing segmentation masks or multi-channel images as inputs. Upon execution, the function opens a graphical user interface with two tabs. In the first tab, hierarchical gating can be performed on expression values stored in the *SingleCellExperiment* object. If the user further provides segmentation masks and (optional) multi-channel images, *cytomapperShiny* visualises expression values and gated cells on images in the second tab. By using the R packages *svglite* and *svgPanZoom*, the *cytomapper* image output is converted to a *scalable vector graphic* which enables pan and zoom functionality. Finally, the user can download gated cells in form of a *SingleCellExperiment* object. For reproducibility purposes, *cytomapperShiny* stores the gates, the gating date and the session information in the *metadata* slot of the downloaded *SingleCellExperiment* object.

S2 CASE STUDY

S2.1 β cell and proinsulin loss during T1D progression

We ranked 100 example images of pancreatic islets (see **Supplementary Note S1.1**) based on the percentage of β cells out of all islet cells and then used the *plotCells* function to visualise islet cell types across all segmentation masks. By labelling the masks based on T1D stage (healthy, recent onset and long-duration), we observe a loss of β cells along disease progression (**Supplementary Fig. 1**).

To validate these results, we ranked multi-channel images based on the mean signal of proinsulin expression (mean pixel-intensity of pixels with a detectable signal, i.e. a signal > 0 counts). We normalised images in a two-step process. First, we performed a min-max scaling to normalise pixel intensities to 0 and 1 across all images. Next, we clipped normalised pixel intensities to 0 and 0.05 removing pixels with high outlying intensities. This approach allows the qualitative comparison of pixel intensities across images. Observing image-to-image differences in total pixel intensities can indicate batch effects in staining efficiency (Somarakis *et al.*, 2019) or biological features such as the expected loss of proinsulin signal over T1D progression (**Supplementary Fig. 2**).

S3 CODE AND DATA AVAILABILITY

All analysis was performed using Bioconductor 3.12, R version 4.0.3 and *cytomapper* version 1.2.0 (available from Github with the tag v1.2.0).

All analysis code and instructions for data analysis are available at:
https://github.com/BodenmillerGroup/cytomapper_publication
The exact version of the scripts is accessible on Zenodo:
https://zenodo.org/record/3994630
A static website visualising the results can be found at:
https://bodenmillergroup.github.io/cytomapper_publication/
A docker container running the exact software used for the analysis can be obtained from: https://hub.docker.com/r/nilseling/bioconductor_cytomapper/tags tag 0.0.3

The release version of the *cytomapper* package can be installed via Bioconductor:
https://www.bioconductor.org/packages/release/bioc/html/cytomapper.html

The development version of the *cytomapper* package:
https://www.bioconductor.org/packages/devel/bioc/html/cytomapper.html or
https://github.com/BodenmillerGroup/cytomapper

The full dataset and the smaller example dataset used in the present publication are available at:
https://data.mendeley.com/datasets/cydmwsfztj/2
Furthermore, the *SingleCellExperiment* object and the *CytoImageList* objects are hosted in the *imcdatasets* package on Bioconductor.

S4 SUPPLEMENTARY FIGURES

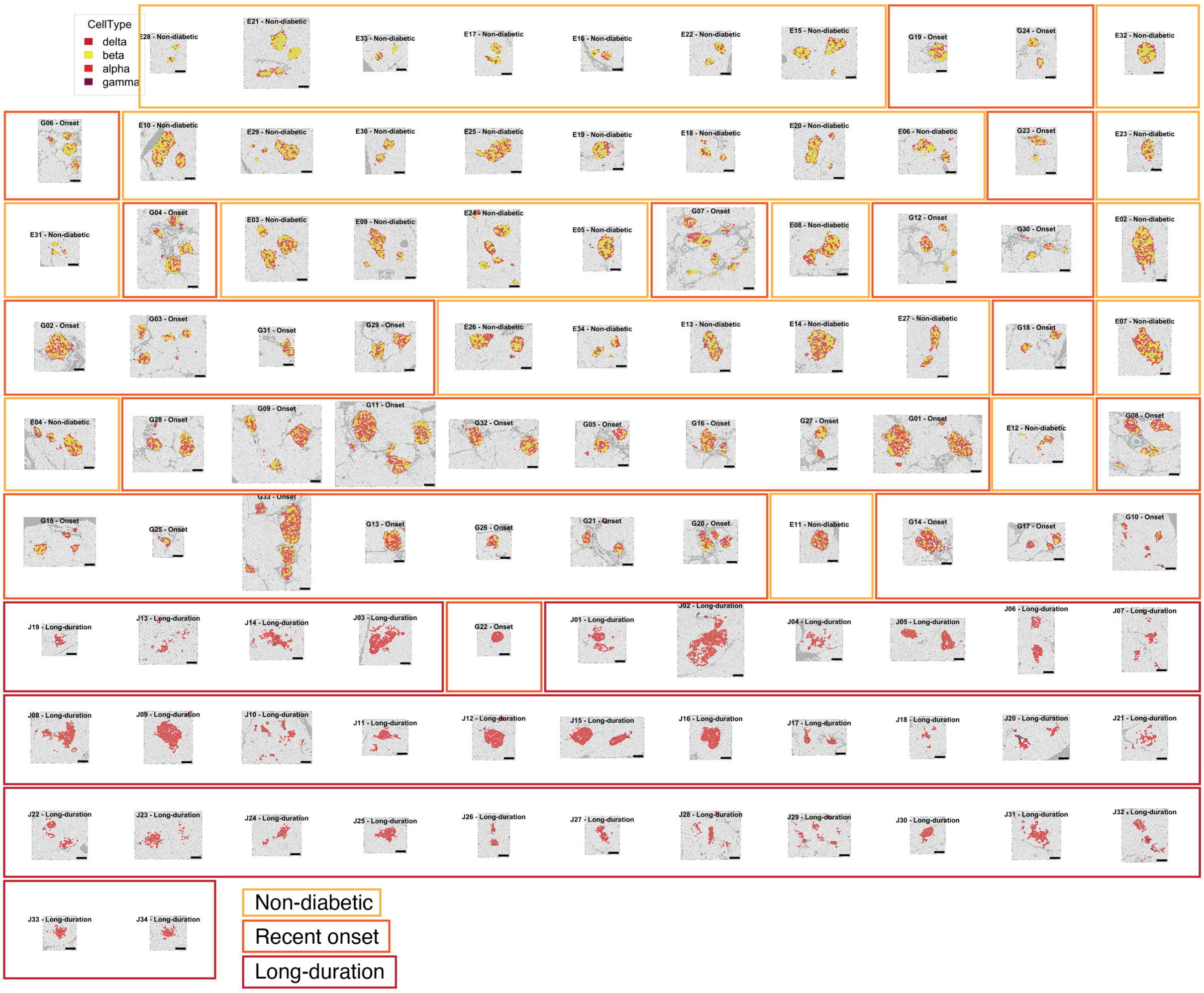

Supplementary Fig. 1: Distribution of islet cell types along T1D progression
A set of 100 segmentation masks was ordered based on the frequency of β cells out of all islet cells. We selected islet cell types by subsetting the *SingleCellExperiment* object; all other cells are displayed in white. The *plotCells* function was used to colour cell areas based on their cell type. Segmentation masks are automatically arranged in a grid-like pattern supporting differences in image dimensions. Image borders are coloured by T1D stage (healthy, recent onset and long-duration). A progressive loss of β cells (coloured in yellow) can be observed. Scale bar: 100µm

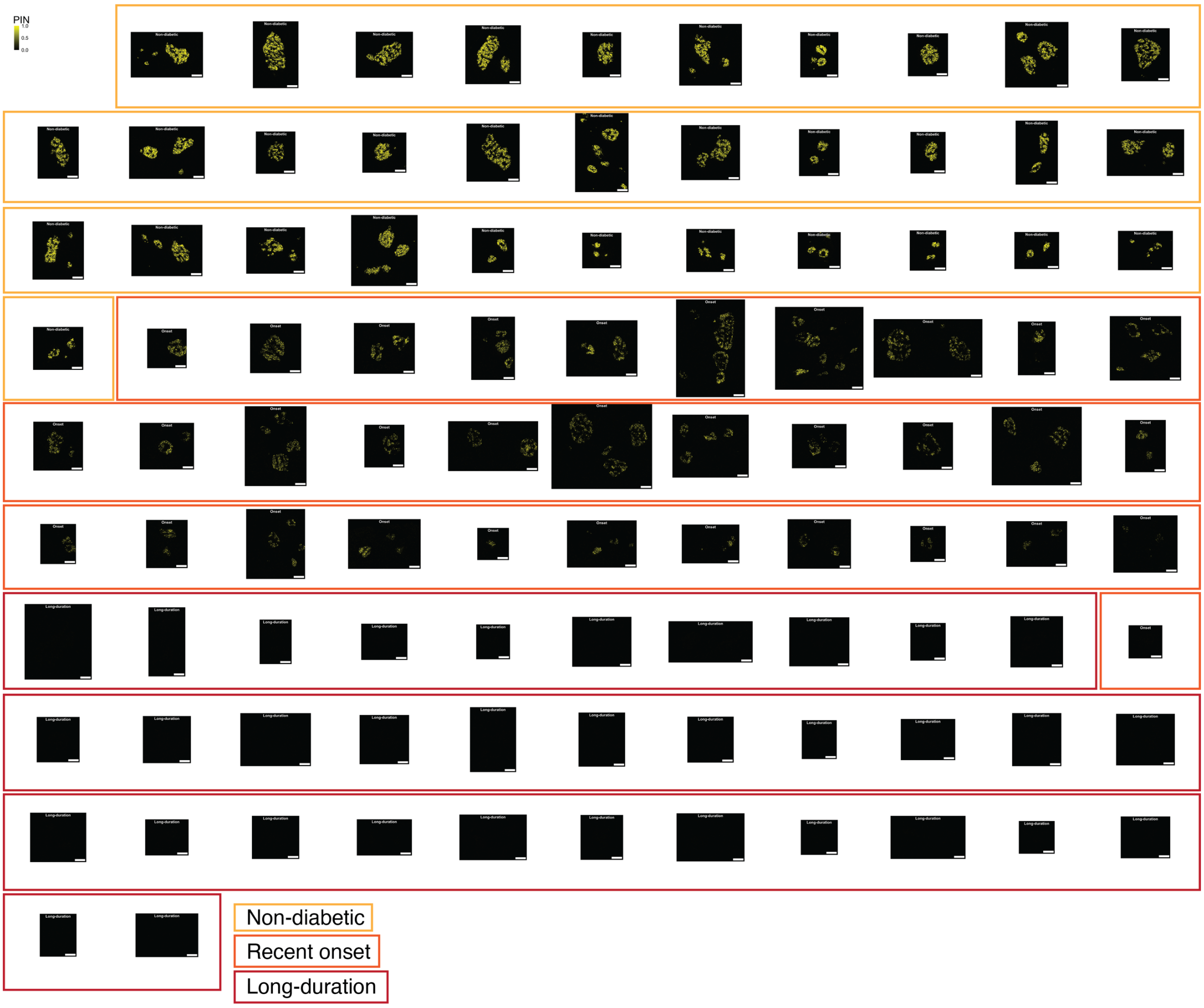

Supplementary Fig. 2: Proinsulin expression across T1D progression

A set of 100 multi-channel images was ordered by calculating the mean of all pixels with detectable proinsulin signal. Images were normalised by first scaling pixel intensities between 0 and 1 across all images. In the second normalisation step, normalised pixel intensities were clipped at 0 and 0.05. Image borders are coloured by T1D stage (healthy, recent onset and long duration). Scale bar: 100µm

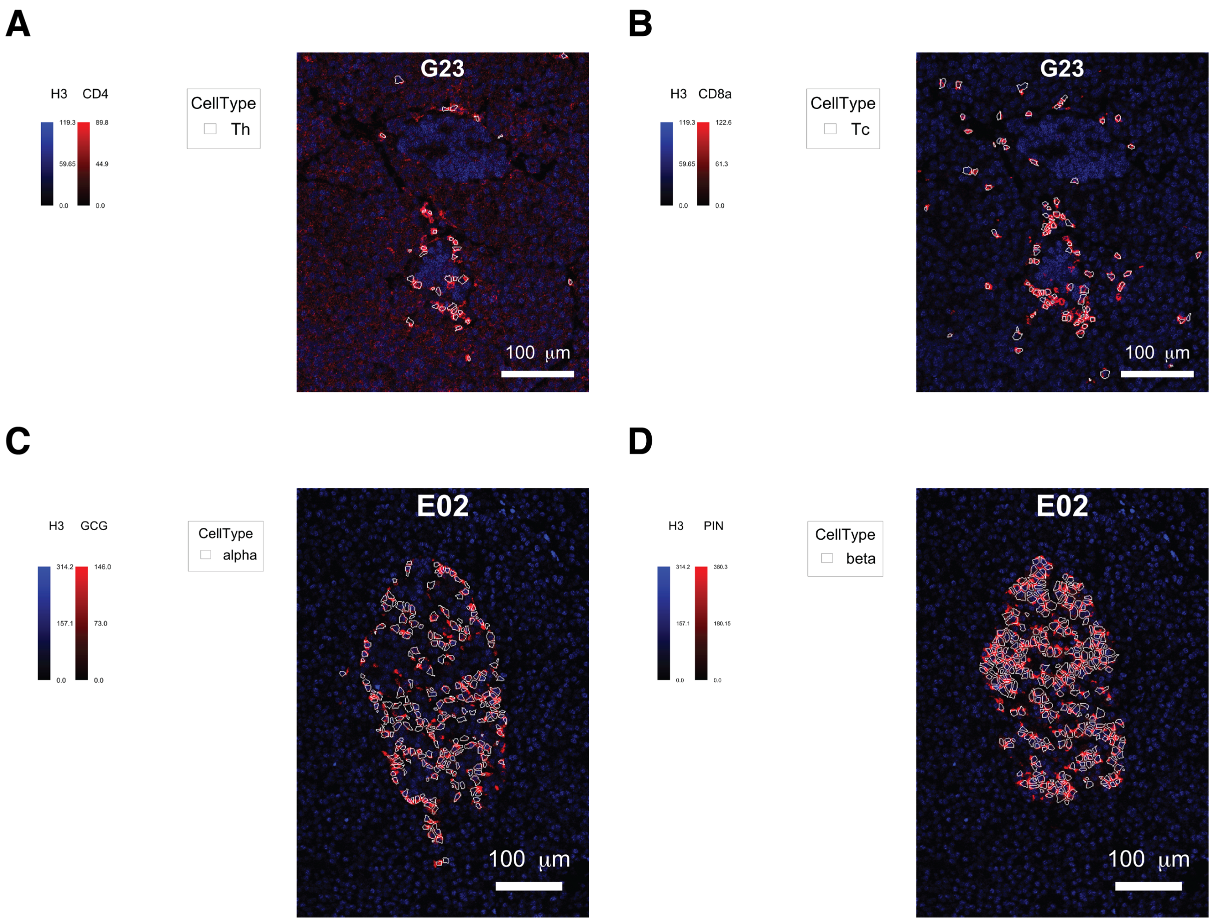

Supplementary Fig. 3: Visual quality control of segmentation and cell-labelling results

(A) – (B) The image with highest T cell density (helper and cytotoxic T cells) was selected. The marker H3 indicates nuclear stain, **(A)** CD4 expression marks helper T cells (Th) and CD8a expression marks cytotoxic T cells (Tc). Individual channels were multiplied by a constant to increase the contrast (H3: 1.5, CD4: 6, CD8a: 6). The *SingleCellExperiment* was subsetted to only contain helper T cells **(A)** or cytotoxic T cells **(B)**. Cells are outlined in white based on their cell type.

(C) – (D) All images of healthy donors were ranked based on their β cell or α cell density. The image with the highest rank sum was selected for visualisation. The marker glucagon (GCG) indicates α cells **(C)** while proinsulin (PIN) is expressed in β cells **(D)**. Individual channels were multiplied by a constant to increase the contrast (H3: 6, GCG: 6, PIN: 6). The *SingleCellExperiment* was subsetted to only contain α cells **(C)** or β cells **(D)**. Cells are outlined in white based on their cell type.

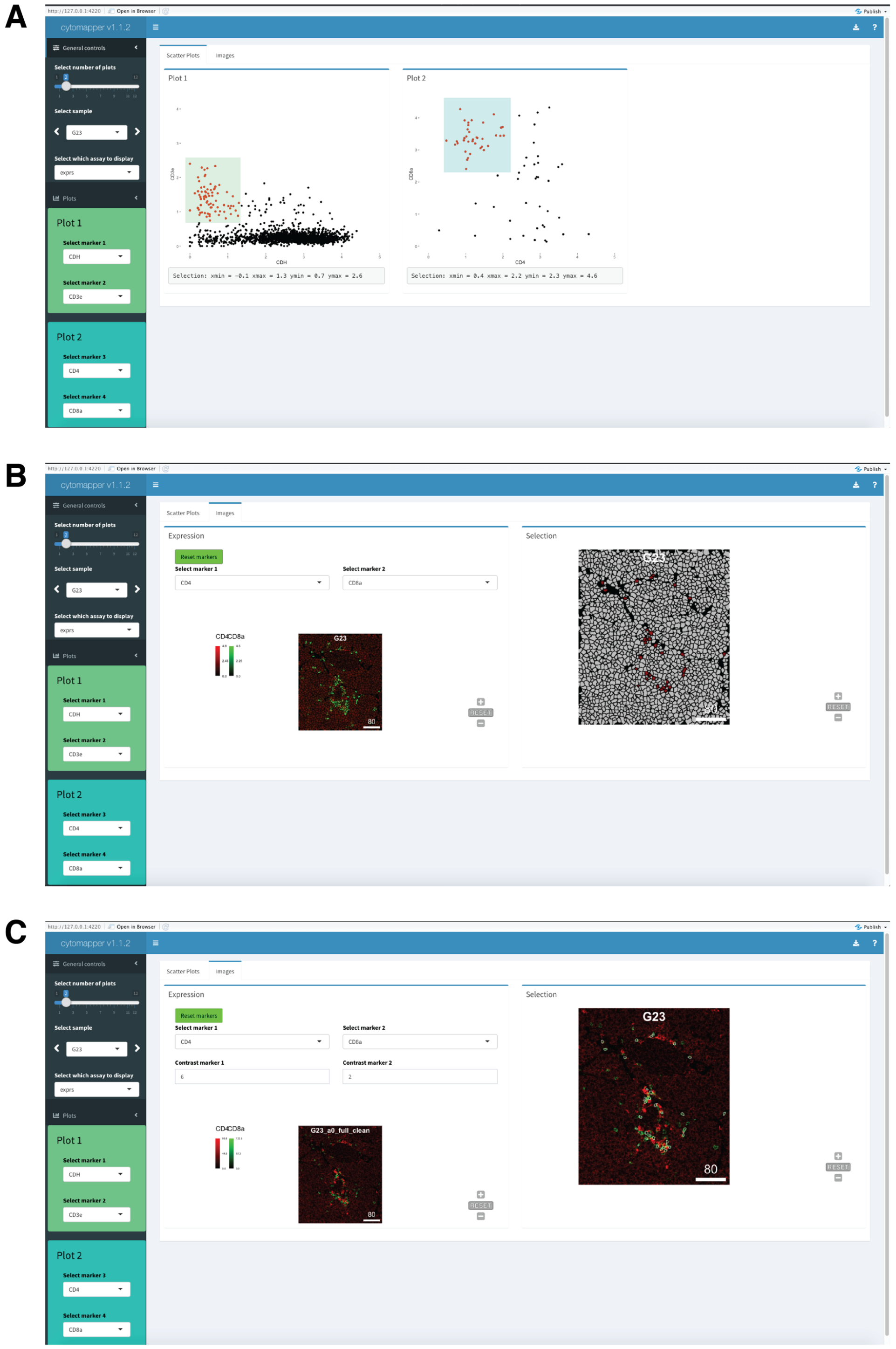

Supplementary Fig. 4: The *cytomapperShiny* GUI

(A) The shiny GUI provided by *cytomapper* allows gating of cells based on their expression. **(B)** If only segmentation masks are supplied, selected cells are coloured as filled objects on their corresponding segmentation mask. **(C)** If the user provides multi-channel images, selected cells are outlined on their corresponding composite image.

Here, we first gated cells based on low E-cadherin (CDH) and high CD3e expression to select T cells. These cells were further sub-gated for high CD8a and low CD4 expression to select cytotoxic T cells.

S5 REFERENCES

Amezquita,R.A. *et al.* (2020) Orchestrating single-cell analysis with Bioconductor. *Nat. Methods*, **17**, 137–145.

Damond,N. *et al.* (2019) A Map of Human Type 1 Diabetes Progression by Imaging Mass Cytometry. *Cell Metab.*, **29**, 755-768.e5.

Kamentsky,L. *et al.* (2011) Improved structure, function and compatibility for cellprofiler: Modular high-throughput image analysis software. *Bioinformatics*, **27**, 1179–1180.

Pau,G. *et al.* (2010) EBImage-an R package for image processing with applications to cellular phenotypes. *Bioinformatics*, **26**, 979–981.

Somarakis,A. *et al.* (2019) ImaCytE: Visual Exploration of Cellular Microenvironments for Imaging Mass Cytometry Data. *IEEE Trans. Vis. Comput. Graph.*, 1–1.

Sommer,C. *et al.* (2011) Ilastik: Interactive learning and segmentation toolkit. *Proc. - Int. Symp. Biomed. Imaging*, 230–233.
